# Predicting undergraduate OSCE performance using traditional and construct-driven situational judgment tests at admission

**DOI:** 10.1007/s10459-024-10379-3

**Published:** 2024-09-30

**Authors:** Ina Mielke, Simon M. Breil, Johanna Hissbach, Maren Ehrhardt, Mirjana Knorr

**Affiliations:** 1 Department of Biochemistry and Molecular Cell Biology, University Medical Center Hamburg-Eppendorf, Hamburg, Germany; 2 Department of Psychology, University of Münster, Münster, Germany; 3 Department of General Practice/Primary Care, University Medical Center Hamburg-Eppendorf, Hamburg, Germany

**Keywords:** Situational judgement tests, Admission, OSCE, Agency, Communion

## Abstract

Situational Judgement Tests (SJTs) are popular to screen for social skills during undergraduate medical admission as they have been shown to predict relevant study outcomes. Two different types of SJTs can be distinguished: Traditional SJTs, which measure general effective behavior, and construct-driven SJTs which are designed to measure specific constructs. To date, there has been no comparison of the predictive validity of these two types of SJTs in medical admission. With the present research, we examine whether the HAM-SJT, a traditional SJT, and the CD-SJT, a construct-driven SJT with an agentic and a communal scale, administered during undergraduate medical admission can predict OSCE (i.e., objective structured clinical examination) performance in a low-stakes (*n*_*LS*_ = 159) and a high-stakes (*n*_*HS*_ = 160) sample of medical students. Results showed a moderate positive relation between the communal scale of the CD-SJT and performance in OSCE stations with trained patients in the high-stakes sample (*r* =.20, *p* =.009). This SJT had also an incremental value in predicting the OSCE performance above and beyond GPA (i.e., grade point average), a science test (i.e., HAM-Nat), and gender (*ß* = 0.18, 95% CI [0.03; 0.33], *p* =.020). That is, individuals who chose more communal behavioral responses in the SJT were rated more favorably in interactions with trained patients in the OSCE. A comparable correlation coefficient was observed for the HAM-SJT when controlling for range restriction due to admission (*r*_*raw*_ = 0.14 vs. *r*_*controlled*_ = 0.20). Our research provides a first indication for the predictive validity of construct-driven SJTs in high-stakes undergraduate medical admission.

**Supplementary Information:**

The online version contains supplementary material available at 10.1007/s10459-024-10379-3.

## Introduction

International consensus holds that successful physicians need social skills alongside other clinical skills and knowledge (Fischer et al., 2015; Frank et al., 2015; General Medical Council, 2018). For this reason, many medical schools already focus on social skills of their future students during the selection process. In undergraduate admission, Situational Judgement Tests (SJTs), which present standardized situations and ask for behavioral responses, have gained popularity as a cost-effective method of screening for social skills in large samples. Historically, they have been developed by experts and the response options represent gradations between more or less effective behaviors, without targeting a specific construct. While these so-called “traditional SJTs” have provided extensive evidence for predicting relevant interpersonal study outcomes such as OSCE (i.e., objective structured clinical examination) performance (see Webster et al., 2020 for a recent meta-analysis), they often lack a thorough understanding of the skills being measured (McDaniel et al., 2016). Over the past decade, an alternative construct-driven approach to SJT development has emerged in personality psychology (Guenole et al., 2017; Lievens, 2017). Here, items are directly related to single predefined constructs and aim to assess individual differences, for example, in compliance (Mussel et al., 2018). Such construct-driven SJTs could improve the theoretical basis for medical student selection (Tiffin et al., 2020). Currently, there is a paucity of studies on construct-driven SJTs used for medical admission, and in particular it is unclear whether they are as valid as traditional SJTs for relevant study outcomes.

### Assessing social skills with traditional and construct-driven SJTs

Social skills can be defined as relatively stable individual capacities that promote effective functioning in interpersonal situations (Breil et al., 2022). The term capacity thus describes an individual’s ability to behave appropriately when the situation requires it. This definition subsumes two crucial conclusions: First, social skills must be seen in the context of specific situations. That is, not every social skill is relevant in every situation but the situational demands determine the relevance of certain skills (Soto et al., 2021). For example, when faced with a patient in distress, physicians with high skill levels of empathy can show understanding and establish a trusting relationship with the patient. When a decision needs to be made by a professional group, physicians with high skill levels of assertiveness can help to structure the discussion and facilitate a decision. Second, social skills are not directly available such as height or weight but are inferred from behavioral information (i.e., how individuals behave in relevant interactions). Behavioral information can be collected, for example, from SJTs which present skill-relevant situations and behaviors. Thereby, SJTs do not measure *actual* behavior in real-life situations or role-plays but *expected* behavior in hypothetical situations. While this basic principle is inherent to so-called traditional and construct-driven SJTs, their developmental process greatly differs (for an overview, see Lievens, 2017; Tiffin et al., 2020).

The aim of traditional SJTs is to mimic samples of previous work experience by creating situations and behavioral responses that reflect reality as closely as possible. Subject matter experts (e.g., physicians) are usually involved in evaluating relevance and appropriateness for the target group. Participants’ responses are usually scored across a significant number of items to obtain a final score. This score broadly reflects a mixture of skills that are relevant to work situations but are often not further specified (Jackson et al., 2017; Schmitt & Chan, 2006). In contrast, the development of construct-driven SJTs mainly follows standard procedures for personality tests and starts with an analysis and definition of relevant skills, usually carried out by test developers who are experts in the construct of interest. Situations are examples of skill-activating situations (e.g., dealing with a patient in distress) and behavioral responses represent different levels of behavior of a social skill (e.g., from low to high empathetic behavior). Work-relevant details are added to create face validity for the target group. Each skill is assessed across a number of items and a score per skill is typically calculated by averaging participants’ responses across corresponding items.

When SJTs have been used to screen for social skills in undergraduate medical admission, they have historically relied on the traditional approach, typically targeting a mixture of key social skills (e.g., Casper in North America or the UKCAT SJT in UK; Dore et al., 2017; Patterson et al., 2017). However, construct-driven SJTs offer great potential as they favor understanding of what is being assessed and may be particularly suitable for selection of undergraduate medical students as they require less work-relevant knowledge than traditional SJTs (Tiffin et al., 2020). From a practitioner perspective, single skill scores allow the definition of differentiated profiles for student selection (e.g., by weighting individual skills scores; Mielke et al., 2022) and offer potential as a feedback tool for medical student training.

### Validation of SJTs with relevant medical study outcomes

An important step in the validation process of SJTs is extrapolation, that is, exploring how a test score relates to real-life performance (Cook et al., 2015; Kane, 2006). For medical students, the real-life domain includes all sorts of potentially critical interpersonal situations during their studies or future working life that require social skills to be successfully managed. Samples of such interpersonal situations are regularly tested during the medical curriculum in objective structured clinical examinations (i.e., OSCEs). Here, medical students are confronted with a series of stations, each simulating a typical medical occasion, and assessors evaluate their performance according to predefined objectives (Harden, 1988). The relevance of social skills can thereby range from low (e.g., applying a suture to a skin model) to high (e.g., psychosomatic history with a simulated patient; Gröne et al., 2022). The final OSCE performance can either be a global score reflecting a mixture of assessed social and other relevant clinical skills, or it can be constructed separately for each predefined social skill (e.g., getting ahead in social situations; Breil et al., 2022).

Several studies in medical admission have demonstrated the value of traditional SJTs in predicting OSCE performance (e.g., Lievens, 2013; Patterson et al., 2013; Patterson et al., 2016). For example, Lievens (2013) reported a corrected correlation of *r* =.15 between a video-based SJT used during undergraduate medical admission and interpersonal OSCE performance nine years after admission. This SJT also had incremental validity in predicting OSCE performance beyond cognitive tests and grade point average (i.e., GPA). Comparable high-stakes evidence is currently lacking for construct-driven SJTs in medical admission, and this may be one reason why they are not used for admission. Most validity studies with construct-driven SJTs are from low-stakes psychological research (e.g., Bledow & Frese, 2009; Mussel et al., 2018; Olaru et al., 2019), and it is unclear whether promising validity evidence can be generalized to high-stakes medical selection.

### Present research

In the present study, we aimed to advance validity research on traditional and construct-driven SJTs in undergraduate medical admission by examining their relations to performance in OSCE stations with and without patient interaction after the third semester and in a low-stakes and a high-stakes context. Further validity evidence on this form of extrapolation is relevant to both research (i.e., do previous findings on construct-driven SJTs from low-stakes research generalize to high-stakes contexts? ) and application (i.e., which SJT predicts relevant study outcomes? ). We used the HAM-SJT (see Schwibbe et al., 2018 for a description of an early version), a traditional SJT designed to screen for a mixture of social skills in undergraduate medical student admission, and the CD-SJT (Mielke et al., 2022), a construct-driven SJT targeting the skills dimensions of agency and communion in a medical study context. Agency and communion skills refer to two distinct social skill dimensions that relate to the underlying core motivations of getting ahead in social situations (agency) and getting along in social situations (communion; see Bakan, 1966; Hogan et al., 1985; Wiggins, 1991). Both factors subsume relevant skills for physicians as mentioned in various international competency frameworks (Breil et al., 2024). That is, agency refers to the ability to demonstrate assertive, decisive, and responsible behavior, whereas communion refers to the ability to demonstrate empathetic, relational, and warm behavior (Mielke et al., 2022). Ideally, physicians have high levels of both skills, which means that they are able to behave either agentic or communal, depending on the needs of the situation.

We collected data during an undergraduate medical admission process, where GPA and a natural science test (i.e., HAM-Nat; Hampe et al., 2008) were crucial cognitive criteria for most of the admitted students. Both criteria have also been shown to predict academic success (Hissbach et al., 2011) and should therefore be included in predictive analyses. The inclusion of cognitive criteria closely mirrors the actual selection process and, in the case of significant predictive validity of SJTs, emphasizes the added value of SJTs over cognitive criteria for the selection of future students (Lievens et al., 2005; Lievens & Patterson, 2011). Another influential factor on interpersonal study outcomes is gender with females sometimes tending to score higher on social skills assessments (Graf et al., 2017; Lievens, 2013).

## Method

### Participants and procedures

We used data from the 2019 and 2020 admission cycles to medical school in Hamburg, Germany. Since legal requirements have changed between the years, relevant differences in the procedures and policies are indicated below.

For the 2019 admission cycle, applicants were invited to the aptitude tests based on a preselection of high school GPA. During the tests, participants completed the HAM-Nat and were asked to voluntarily complete the HAM-SJT and the CD-SJT. Both SJTs were part of a research study within the STAV project (Studierendenauswahl-Verbund, 2020) and participants were aware that their performance would not contribute to their admission decision (i.e., low-stakes condition), but received a detailed performance feedback. After consenting to STAV, participants answered several sociodemographic questions.

For the 2020 admission cycle, there was no preselection and all registered participants were invited to take the aptitude tests. Participants completed the HAM-SJT and the CD-SJT under identical high-stakes conditions. This year, the HAM-SJT contributed to selection decisions within the largest selection quota, responsible for 60% of the available study places (weighting: 20% HAM-SJT, 40% GPA, 40% HAM-Nat). The CD-SJT was included for research purposes only. Participants knew that their SJT performance influenced their admission decision but they were not aware that only the HAM-SJT was relevant. In exchange for detailed performance feedback, they were also asked to provide consent for the STAV project and sociodemographic information.

After the third semester, students completed a mandatory OSCE as part of an interim exam. The OSCE consisted of ten stations, each lasting five minutes, and each station was assessed by an expert in the field. Each station focused on a specific topic (e.g., medical history taking) and tested basic clinical skills, including social (e.g., active listening) and practical (e.g., blood sampling) skills.

**Fig. 1:**
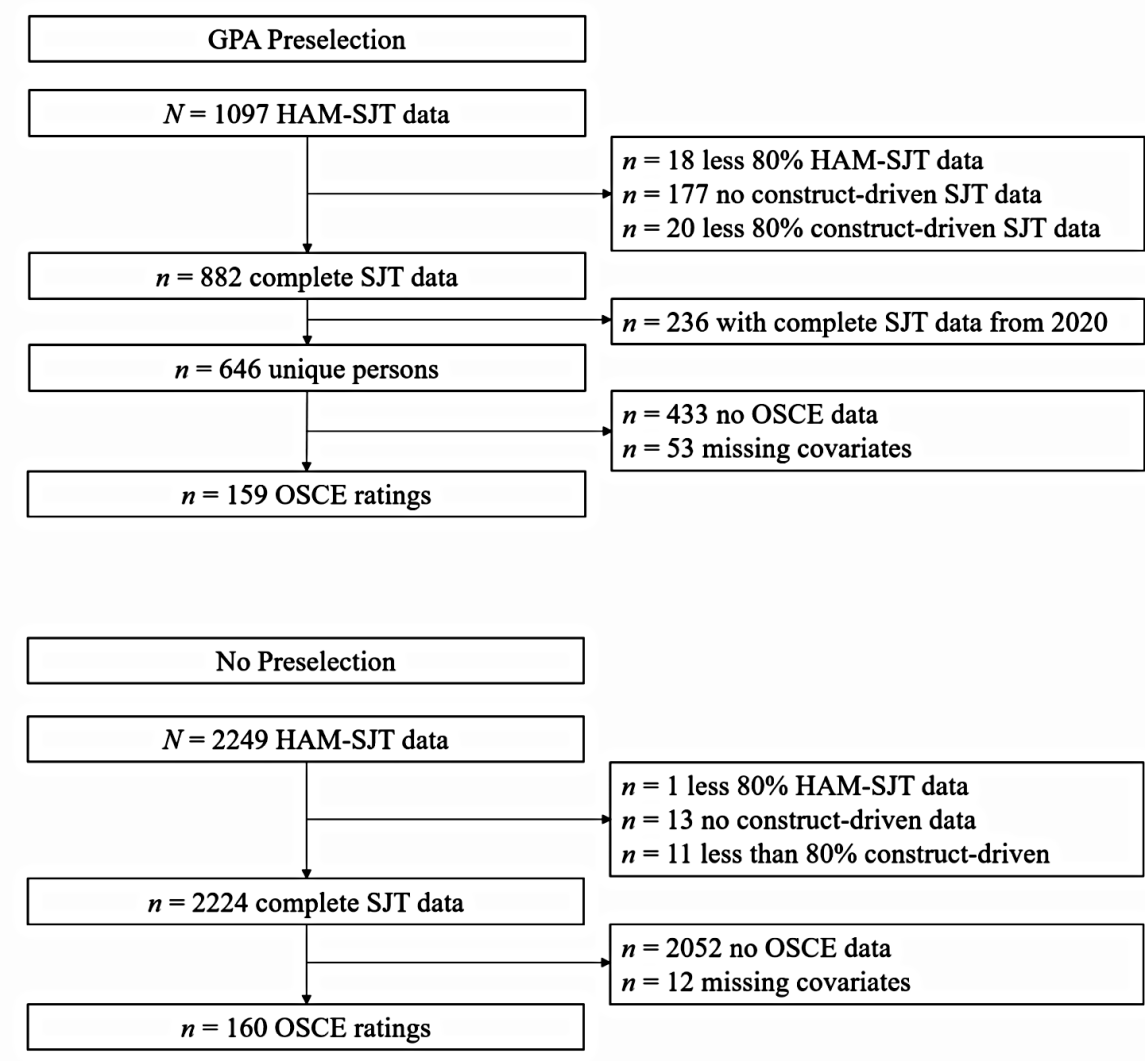
Flowchart of detection for low-stakes (top) and high-stakes sample

In 2019 and 2020, *N*_*LS*_ = 1097 and *N*_*HS*_ = 2249 applicants consented to STAV and participated in the aptitude tests. To ensure a conscientious completion, we excluded participants who completed less than 80% of the HAM-SJT or one CD-SJT scale, reducing the samples to *n*_*LS*_ = 882 and *n*_*HS*_ = 2224^1^. If applicants participated in 2019 and 2020, we selected the more recent data from 2020 which reduced the 2019 sample to *n*_*LS*_ = 646 (additional analyses for the 2020 sample with 2019 SJT data can be found in the Supplement). In a next step, we matched the SJT data to the outcome data and determined samples with complete SJT, OSCE, and covariate data (see Fig. 1). The final samples consisted of *n*_LS_ = 159 (69% female; mean age 20.23 years with *SD =* 1.55) and *n*_*HS*_ = 160 (61% female; mean age 21.00 years with *SD =* 2.60).

### Measures

#### HAM-SJT

**Fig. 2:**
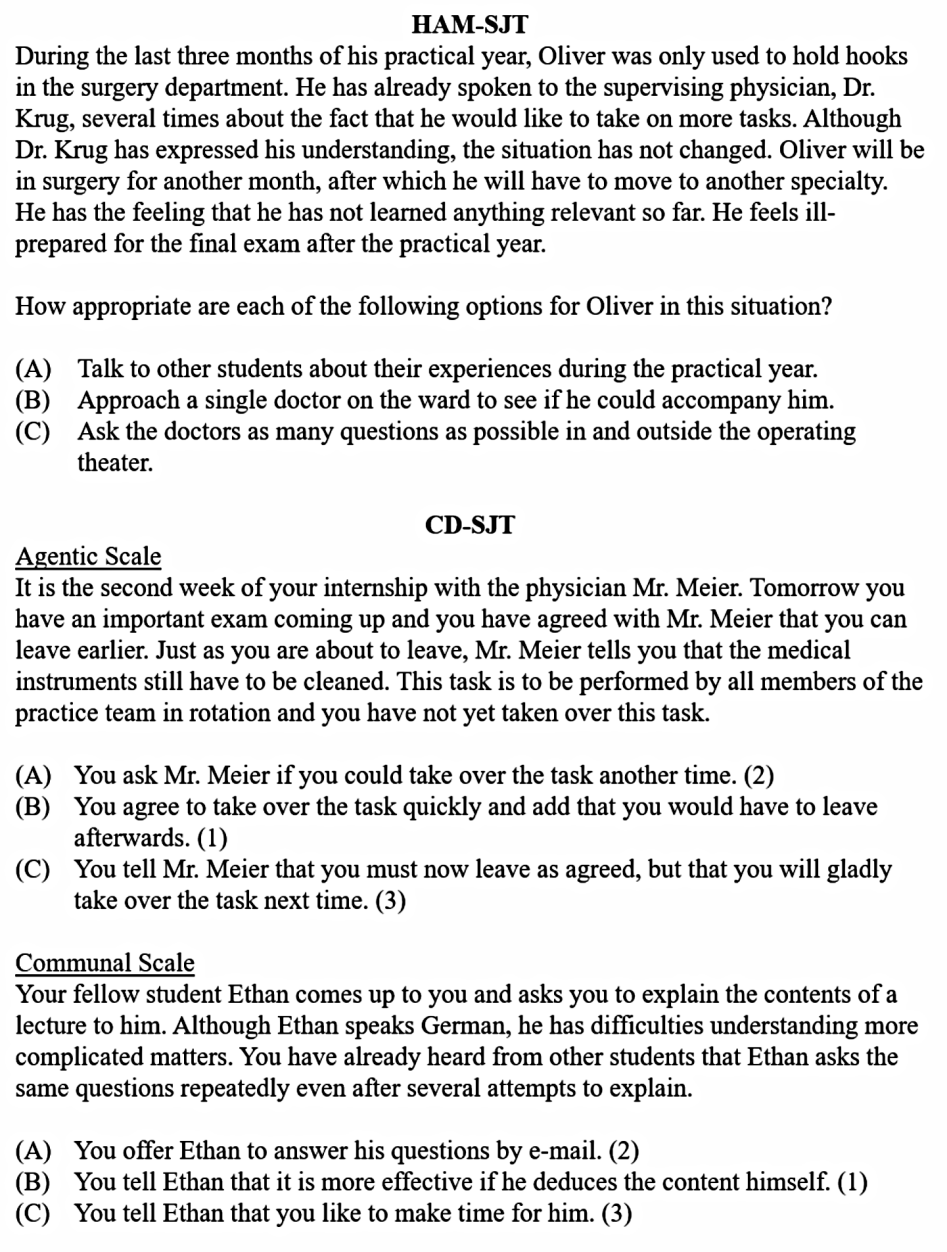
Example items for all SJTs with construct level for CD-SJT in parentheses

The situations and behavioral responses in the HAM-SJT were developed by personality psychologists and reviewed by subject matter experts (SMEs; Schwibbe et al., 2018). Situations describe typical encounters of a medical student with fellow students, patients, patients’ relatives, physicians, or medical staff during teaching, practical training, or other study occasions. The main character is always confronted with some kind of problem or dilemma and has to decide how to react (see Fig. 2 for an example), but no specific construct is targeted at the situation or item level. The HAM-SJT consisted of 18 situations with 86 behavioral responses in 2019 and 20 situations with 80 behavioral responses^2^ in 2020. Although the specific content changed between years, the blueprint remained stable, including development steps, test instructions, context and structure of scenarios and behavioral responses, and, in part, critical incidents. In 2020, there were three test versions of the HAM-SJT with parallel items that were based on the same critical incident (Lievens & Sackett, 2007) and similarly rated by a panel of SMEs. Participants were asked to rate the appropriateness of each behavioral response for the main character on a scale from *1* (very appropriate) to *4* (very inappropriate). Participants’ responses were intraindividually *z*-standardized to account for different response styles (McDaniel et al., 2011) and compared to a panel of SME by averaging the sum of the squared difference from the intraindividually *z*-standardized expert mean. The resulting score was linearly transformed by subtracting it from the highest occurring score such that the higher the participant’s score, the lower the deviation from the SME panel.

#### CD-SJT

The same personality psychologists developed situations and behavioral responses for the CD-SJT and other research team members reviewed the construct fit (see Mielke et al., 2022 for a detailed description). Situations are written from a first-person perspective and describe similar encounters to the HAM-SJT, although in less detail. Each situation intentionally includes a trigger to activate either agency or communion and a dilemma that argues against highly agentic or communal behavior (see Fig. 2 for an example and the codebook in the OSF project https://osf.io/d7mb6/). For each construct, 15 situations with three behavioral responses reflecting different levels of agentic or communal behavior were included. In 2019, a pilot version of the construct-driven SJT was used, which then underwent a final review afterwards. Most of the critical incidents were retained (63%), and changes mainly involved adjusting the trigger and dilemma of the existing situations and rewording of the behavioral responses to increase response variability. Participants were asked to indicate the behavioral response that best described their likely behavior in the situation. Their responses were recoded according to the construct level on a scale from *1 (lowest degree of construct corresponding behavior)* to *3 (highest degree of construct corresponding behavior)* and a final score was calculated by averaging across the 15 responses per construct. In 2020, a subsample (*n* = 63) received a slightly different version that included ten situations per construct and a ranking instruction that asked participants to sort the three behavioral responses according to the likelihood of their own behavior. Items were selected based on their factor loadings for the respective construct. Here, we recoded the first ranked behavioral response according to the construct level of that response and averaged across the 10 responses per construct (additional analyses for the different instructions can be found in the Supplement). The higher the final score, the more the participant tended to respond to interpersonal situations with agentic or communal behavior.

#### OSCE performance

The OSCE stations were not designed with a construct-driven focus but varied in the extent to which social skills were relevant in addition to practical skills. Accordingly, we distinguished between OSCE performance in stations with interaction with a trained patient (i.e., *OSCE Interaction*) and OSCE stations without such interaction (i.e., *OSCE Other*). Interactions included taking medical history or performing a physical examination. With the exception of one station, the OSCE was identical in the low-stakes and the high-stakes sample. The OSCE Interaction score comprises performance in six (low-stakes sample) or seven (high-stakes sample) of the ten stations. Each OSCE station was scored by a field expert using checklist items describing specific actions to be taken in the situation. If a student carried out the action according to the content of the item, he or she received the assigned points. Per station, all items added up to between 0 and 20 points. The OSCE Interaction and OSCE Other scores were calculated by averaging the points of the respective stations.

We had originally planned to analyze how the CD-SJT in the low-stakes sample is related to a single OSCE station on basic communication skills (see https://osf.io/bs8uz). However, due to COVID restrictions at the time, this OSCE station was excluded for the present samples and we decided to use the OSCE performance scores instead.

#### Covariates

Participants’ GPA reflects a complex aggregate of performance across different subjects and exams during the last two or three years of high school. The original grade was linearly transformed on a scale from *0 (lowest possible grade)* to *30 (highest possible grade)*. The HAM-Nat consisted of 78 (2019) and 60 (2020) single-choice natural science questions with five possible answers. Participants’ responses were coded as *0 (incorrect answer)* or *1 (correct answer)* and a final score was calculated by averaging across all test items. Gender was coded as 0 if participants identified as male and as 1 if they identified as female.

### Statistical analysis

Data preparation and analysis were performed using R version 4.0.1 (R Core Team, 2020). As a first step, we computed Pearson correlations and an additional correlation that corrected for the range restriction in the HAM-SJTs due to the admission decision in the high-stakes sample (see Patterson et al., 2017 for a similar approach). For the correction, we used Thorndike’s Case 2 (Stauffer & Mendoza, 2001) and derived the unrestricted standard deviation from the dataset before matching the SJT to the OSCE and covariate data, thus including both admitted and non-admitted participants. We then computed a baseline regression model for each cohort in which all covariates (i.e., GPA, HAM-Nat, and gender) were regressed on the OSCE Interaction performance^3^. To estimate the effect of SJT performance, this model was extended to include performance on either the HAM-SJT, or the agentic or the communal scale of the CD-SJT. We refer to a significance level of α =.05 for all analyses and to correlations around.10 as being small, around.20 as being medium, and above.30 as being large (Gignac & Szodorai, 2016; Lovakov & Agadullina, 2021).

## Results

In Table 1, we present descriptive statistics, McDonalds Omega as a reliability coefficient, and correlations separately for both cohorts.

**Table 1:** Descriptive statistics, reliability coefficients, and correlations

	Low-Stakes			High-Stakes																			
	*M*	*SD*	Rel	*M*	*SD*	Rel	1.	2.		3.		4.		5.		6.		7.		8.		9.	
1. HAM-SJT	0.34	0.12	0.67	0.29	0.10	0.89/0.85/0.87	-	− 0.01		**0.26**		0.14 (0.20)		0.09 (0.13)		− 0.08		0.02		0.11		0.04	
2. CD-SJT Agency	2.39	0.21	0.58	2.08	0.24	0.43/0.69	0.09	-		**− 0.22**		− 0.04		0.02		− 0.01		− 0.03		0.12		− 0.05	
3. CD-SJT Communion	2.45	0.21	0.62	2.69	0.21	0.64/0.54	**0.16**	− 0.07		-		**0.20**		0.10		0.09		0.03		0.06		− 0.02	
4. OSCE Interaction	17.18	1.14	0.69	17.19	0.99	0.60	0.05	− 0.08		0.04		-		**0.32**		**0.22**		− 0.05		**0.20**		− 0.14	
5. OSCE Other	17.94	1.01	0.17	17.73	1.14	0.27	0.00	− 0.09		− 0.03		**0.40**		**-**		**0.20**		− 0.03		**0.24**		− 0.02	
6. GPA	25.50	2.15	-	23.98	3.60	-	− 0.01	0.06		0.08		0.13		0.08		-		− 0.05		**0.19**		**− 0.66**	
7. HAM-Nat	0.66	0.10	0.81	0.72	0.15	0.93/0.91/0.90/-	**− 0.17**	− 0.03		**− 0.23**		0.01		0.05		**− 0.16**		-		**− 0.21**		− 0.11	
8. Gender	0.69	0.46	-	0.61	0.49	-	**0.17**	0.05		**0.17**		**0.22**		0.08		− 0.06		− 0.14		-		− 0.12	
9. Age	20.23	1.55	-	21.00	2.60	-	− 0.03	− 0.04		− 0.01		− 0.10		**− 0.18**		**− 0.42**		**0.14**		− 0.01		-	

*Note*n _LS_ = 159 and *n*_HS_ = 160. Reliability for OSCE was based on station points. In 2020, there were three parallel test versions of the HAM-SJT, two versions of the CD-SJT, and four versions of the HAM-Nat. For one version of the HAM-Nat, the subsample was too small to calculate a reliability score. Correlations in the lower triangle for the low-stakes cohort and in the upper triangle for the high-stakes cohort. Correlation in parentheses was corrected for range restriction on the HAM-SJTs according to Thorndike’s Case 2 (Stauffer & Mendoza, 2001). Gender was coded as 0 = male and 1 = female. Bold coefficients indicate significant results at *p* <.05

In the low-stakes sample, individual correlations between OSCE Interaction or OSCE Other and the HAM-SJT (*r* =.05, *p* =.547 and *r* =.00, *p* =.969), the agentic scale of the CD-SJT (*r* = −.08, *p* =.340 and *r* = −.09, *p* =.237), or the communal scale of the CD-SJT (*r* =.04, *p* =.619 and *r* = −.03, *p* =.686) remained insignificant. Higher performance on the HAM-SJT and on the communal scale of the CD-SJT were associated with lower performance on the HAM-Nat (*r* = −.17, *p* =.028 and *r* = −.23, *p =*.004) and female gender (*r* =.17, *p* =.031 and *r* =.17, *p* =.032), respectively. Both SJTs (*r* =.16, *p* =.040) and both OSCE scores (*r* =.40, *p* <.001) were positively related.

In the high-stakes sample, higher performance on the communal scale of the CD-SJT was associated with better OSCE Interaction performance (*r* =.20, *p* =.009) but not with better OSCE Other performance (*r* =.10, *p* =.190). Correlations between OSCE Interaction or OSCE Other performance and the agentic scale of the CD-SJT (*r* = −.04, *p* =.646 and *r* =.02, *p* =.850) or the HAM-SJT (*r* =.14, *p* =.083 and *r* =.09, *p* =.252) remained insignificant. However, when controlling for the range restriction on the HAM-SJT due to its relevance in one admission quota, the relation between the HAM-SJT and OSCE Interaction performance increased to *r* =.20, which was comparable to the moderate effect found for the communal scale of the CD-SJT. The communal scale was positively related to the HAM-SJT (*r* =.26, *p* <.001) and negatively related to the agentic scale (*r* = −.22, *p* =.004). Both OSCE scores were again positively related (*r* =.32, *p* <.001).

The positive relation between the communal scale of the CD-SJT and OSCE Interaction performance in the high-stakes sample remained significant when controlling for GPA, HAM-Nat, and gender (*ß* = 0.18, 95% CI [0.03; 0.33], *p* =.020; see Table 2). That is, participants who tended towards warm behavior in the CD-SJT also performed better across OSCE stations with trained patients, independent of cognitive criteria and gender.

**Table 2:** Summary of multiple regression analyses of prediction on OSCE Interaction performance

	Low-Stakes Sample						High-Stakes Sample				
Predictor	*ß*	95% CI	*p*	adj. *R*²	Δ*R*²		*ß*	95% CI	*p*	adj. *R*²	Δ*R*²
Step 1											
GPA	0.15	[0.00; 0.31]	0.051	0.055**			0.19	[0.03; 0.34]	0.019*	0.057**	
HAM-Nat	0.07	[-0.09; 0.22]	0.412				-0.01	[-0.16; 0.15]	0.907		
Gender	0.24	[0.08; 0.39]	0.003**				0.17	[0.01; 0.33]	0.039*		
Step 2a											
HAM-SJT	0.02	[-0.14; 0.18]	0.787	0.049*	− 0.006		0.14	[-0.01; 0.29]	0.074	0.070**	0.013
Step 2b											
CD-SJT Agency	-0.10	[-0.25; 0.06]	0.215	0.058*	0.003		-0.06	[-0.21; 0.10]	0.462	0.054*	− 0.003
Step 2c											
CD-SJT Communion	0.00	[-0.16; 0.16]	0.984	0.048*	− 0.006		0.18	[0.03; 0.33]	0.020*	0.084**	0.027*

*Note* Regression weights for predictors from Step 1 changed slightly when other predictors were added (see the Supplement for detailed summaries). Predictors in Step 2 were each added separately to Step 1, not combined. ΔR² compared to the Step 1 model. Gender was coded as 0 = female and 1 = male. * *p* <.05. ** *p* <.01

## Discussion

The conceptualization and assessment of social skills is still a hot topic in medical education, for example in the selection of promising future students. While research on the validity of construct-driven SJTs screening for social skills is a vibrant subject in psychology, it remains a blind spot in applied medical education so far. With the present paper, we contribute to the emerging research by introducing initial evidence for a relevant relationship between a construct-driven SJT and OSCE performance. Specifically, we aimed to assess whether a construct-driven SJT was equally suitable than a traditional SJT in predicting OSCE performance after the third semester. Results show that the communal scale of the CD-SJT was positively related to OSCE Interaction performance in the high-stakes context and predicted the OSCE performance when controlling for cognitive criteria and gender. Relations between the HAM-SJT or the agentic scale of the CD-SJT and OSCE performance scores remained insignificant. However, when correcting for range restriction due to the admission decision, the HAM-SJT and the communal scale of the CD-SJT showed equally high correlations with OSCE Interaction performance.

The positive relation between the communal scale of the CD-SJT and OSCE Interaction performance in the high-stakes sample marks a conceptual overlap between the two assessments, despite their methodological differences. While participants in the CD-SJT indicated their *intention to behave* empathetically, compassionately, and kindly when the situation called for it, OSCE Interaction performance included ratings of *actual behavior* in real (though staged) situations with trained patients, some of which called for communal behavior. Those who self-reported a higher communal skill on the SJT were also able to translate this intention into observable actions during relevant OSCE stations. The relation is remarkable when considering the time lag of almost two years between the completion of the SJT before admission and the implementation of the OSCE, and the personal development some students may have undergone since the beginning of their medical studies (Abbiati & Cerutti, 2023; Deventer et al., 2019). Neither cognitive ability nor gender differences explained the unique association between CD-SJT and OSCE performance, supporting the incremental potential of such a construct-driven SJT as an additional criterion for undergraduate medical admission. Compared with previous evidence on the predictive validity of SJTs for medical admission, the present effect size of *r* =.20 is similar to the pooled estimate of *ß* = 0.32 that was previously reported for traditional SJTs and tended to be significantly smaller for undergraduate medical selection (Webster et al., 2020). The absence of a significant relation to OSCE performance in stations without interactions marks an argument towards the discriminant validity of the CD-SJT. That is, the communal scale of the CD-SJT only predicts performance when situations include an interactional potential and offer the possibility to behave empathetic towards a patient.

In contrast to the CD-SJT, the traditional HAM-SJT was indeed relevant for admission in the high-stakes sample. When controlling for the range restriction introduced by the admission decision, the strength of the relation between the HAM-SJT and OSCE Interaction performance was similar to the communal scale of CD-SJT, though the raw correlation was insignificant. As the HAM-SJT and the communal scale of the CD-SJT only moderately converge, the HAM-SJT and OSCE Interaction performance appear to overlap on other relevant skills than communion. However, due to the traditional developmental approach which combines different skills at the item and test score level, we cannot clearly state which other constructs in the HAM-SJT led to the positive relation to OSCE performance. This highlights a limitation of the traditional approach that the construct-driven approach seeks to overcome.

The agentic scale of the CD-SJT remained unrelated to OSCE performance scores in both samples. One reason may be the irrelevance of assertive, responsible, and leadership behavior for students in the early years of study. That is, the ability to behave agentic becomes an important social skill for physicians only after graduation, for example in decision-making with colleagues, in emergency situations, or in patient education. Thus, although a tendency to agentic behavior was reported, this tendency could not be observed in early OSCE performance because agency was irrelevant in most OSCE situations. An alternative explanation may be the inability of the CD-SJT to assess participants’ agentic skill. Participants with high agentic skills may not have identified all the triggering situations that could be successfully managed with more agentic behavior. This may also have led to the low internal consistency of the agentic scale in the high-stakes sample. Therefore, the present result for the agentic scale is not a general argument against the importance of physicians’ agentic skills, but opens a discussion for their definition and assessment.

While there were significant relations between the SJTs and OSCE Interaction performance in the high-stakes sample, there were no comparable results in the low-stakes sample. Here, participants completed the SJTs after taking the HAM-Nat and knew that their SJT performance had no influence on the admission decision. Accordingly, their effort may have decreased, resulting in less conscientious completion. Content of both SJTs also differed slightly between the low-stakes and high-stakes samples, and the lack of significant relations with OSCE performance may be partly due to unintended changes in content and assessed constructs. However, we used the HAM-SJT in four low-stakes and five high-stakes years with different test content in each year, and our results show that average performance increased drastically in high-stakes years since the HAM-SJT was used for selection purposes (*t*(6944.90) = 48.54, *p* <.001, *d* = 0.92 with pooled means and standard deviations). There are, nevertheless, differences in content and one could argue that we have rather two HAM-SJT and two CD-SJT versions. In this case, our results still show that one construct-driven SJT was able to predict OSCE Interaction performance.

### Limitations and future directions

We assume that the differences in results between the low-stakes and high-stakes samples are mainly due to the different test conditions, which influenced how conscientiously and honestly participants completed the SJTs. However, we were not able to statistically disentangle effects of test content from effects of test conditions and therefore cannot completely rule out the possibility that results between samples were also influenced by different versions of the SJT. Although our findings are still meaningful, future studies focusing on the influence of test conditions are advised to remedy this shortcoming by using the same SJT test version in a low-stakes and a high-stakes sample. If one is interested in creating parallel test versions, the relationship between SJTs and study outcomes can be examined with constant test conditions and changing SJT versions.

We used a construct-driven SJT with an agentic and a communal scale and a traditional SJT assessing a mixture of social skills. The findings differed between the SJTs, suggesting varying results depending on the skills assessed. Our selection is only one example of relevant social skills and future research is encouraged to replicate the current findings using alternative SJTs, particularly construct-driven SJTs that focus on relevant constructs other than agency and communion (e.g. skills not exclusively in the social domain such as self-management skills; Soto et al., 2021). Similar to our traditional SJT, the present OSCE focused on clinically relevant tasks requiring a mixture of social and practical skills. Although we distinguished between stations with and without interactions with trained patients, the tasks and checklists included not only social skills but also practical skills that have no communication content. To improve the understanding of the constructs assessed in existing OSCEs, researchers could use performance ratings on single stations or items with interpersonal content only rather than OSCE stations with mixed focus (Gröne et al., 2022). In our case, however, performance was only assessed by a single rater per station and stations solely focusing on social skills were reduced due to COVID restrictions at the time. When OSCEs are newly designed or can be adapted, it is recommended to assess one skill per station and multiple stations per skill to increase reliability (Breil, Forthmann et al., 2022).

In addition to the developmental approach, one design feature that differed between the HAM-SJT and the CD-SJT was the response instruction. Whereas participants in the HAM-SJT were asked to indicate the appropriateness of each behavioral response (i.e., knowledge instruction), the CD-SJT used a behavioral tendency instruction in which participants selected the option that most resembled their own behavior. Meta-analysis has shown that SJTs with knowledge instructions are more likely to correlate with cognitive ability than SJTs with behavioral tendency instructions which rather correlate with other personality constructs (McDaniel et al., 2007). Accordingly, the constructs measured in our SJTs may have been influenced not only by the developmental approach but also by the response instruction. Yet, the two SJTs did not differ in their relationships with GPA and HAM-Nat performance as two indicators of cognitive ability. Furthermore, given that the type of response instruction was not a moderator of criterion validity in the mentioned meta-analysis, we do not believe that it influenced the relationship with OSCE performance. Future research can investigate this further and disentangle response instruction from developing approach by using both response instruction for a traditional and a construct-driven SJT.

Our SJTs were administered in addition to a well-established on-site aptitude test, and we therefore used the same classic paper-pencil format. Although such written SJTs are easier to develop and administer, video-based SJTs provide a more realistic picture of situations and, consequently, lead to higher predictive validity of interpersonal outcomes (Lievens & Sackett, 2006). If researchers and practitioners are interested in approaching applicants’ actual behavior more closely, they could switch from closed to open-ended written response formats (e.g., Dore et al., 2017) or even ask applicants to videotape their reactions to the presented situations and rate the reactions afterwards (e.g., with respect to agency or communion). However, the higher the fidelity of SJTs, the more resources are needed (e.g., videos, raters), which quickly reaches its limits in admission processes with large numbers of applicants.

## Conclusion

In summary, the present findings are consistent with previous evidence on the prediction of interpersonal study outcomes by SJTs used for undergraduate medical admission. More importantly, this is the first study to show that a construct-driven SJT focusing on communal skills is at least equally suitable than a traditional SJT at predicting OSCE performance beyond cognitive criteria and gender. These results highlight the potential of construct-driven SJTs to supplement undergraduate medical admission with a theory-based assessment of social skills that is scalable to large numbers of participants. However, before construct-driven SJTs are used for admission decision, further research on the generalizability and feasibility (e.g., faking or creation of parallel test versions; Kasten et al., 2020; Lievens & Sackett, 2007) is recommended.

## Electronic supplementary material

Below is the link to the electronic supplementary material.

Supplementary Material 1

## Data Availability

Data can be provided via an official data request file available in the OSF.

## Footnotes

1 If we would not have controlled for 80% item completion, the final samples would have been *n*_*LS*_ =161 and *n*_*HS*_ = 161.

2 The test included two additional situations and six additional behavioral responses for research purposes, which were not used to calculate the final score.

3 We did not repeat the regression analysis with OSCE Other performance as we did not observe any significant correlations to SJT performance.
