# Supplementary material for: Predicting undergraduate OSCE performance using traditional and construct-driven situational judgment tests at admission
Source: Adv Health Sci Educ Theory Pract. 2024 Sep 30;30(3):777–94. doi: 10.1007/s10459-024-10379-3 (PMC12119639; doi:10.1007/s10459-024-10379-3)
Supplement: Supplementary file 1 — Supplementary Material 1 [file 10459_2024_10379_MOESM1_ESM.docx]

**Predicting Undergraduate OSCE Performance Using Traditional and Construct-Driven Situational Judgment Tests at Admission**

*Advances in Health Sciences Education*

Ina Mielke^1^ (ORCID: 0000-0003-1764-5553)

Simon M. Breil^2^ (ORCID: 0000-0001-5583-3884)

Johanna Hissbach^1^

Maren Ehrhardt^3^

Mirjana Knorr^1^ (ORCID: 0000-0002-0996-9286)

^1^Department of Biochemistry and Molecular Cell Biology, University Medical Center Hamburg-Eppendorf, Germany

^2^Department of Psychology, University of Münster, Germany

^3^Department of General Practice / Primary Care, University Medical Center Hamburg-Eppendorf, Germany

Correspondence concerning this article should be addressed to Ina Mielke ([i.mielke@uke.de](mailto:i.mielke@uke.de))

**Detailed Model Summaries for Main Regression Analyses**

**Table S1** Summary of Multiple Regression Analyses of Prediction on OSCE Interaction Performance for Low-Stakes Sample

| Variable | Step 1 | Step 2a | Step 2b | Step 2c |
| --- | --- | --- | --- | --- |
| GPA | 0.15 [0.00; 0.31] | 0.15 [0.00; 0.31] | **0.16** [0.00; 0.32] | 0.15 [0.00; 0.31] |
| HAM-Nat | 0.07 [-0.09; 0.22] | 0.07 [-0.09; 0.23] | 0.06 [-0.09; 0.22] | 0.07 [-0.09; 0.23] |
| Gender | **0.24** [0.08; 0.39] | **0.23** [0.08; 0.39] | **0.24** [0.09; 0.40] | **0.24** [0.08; 0.39] |
| HAM-SJT |  | 0.02 [-0.14; 0.18] |  |  |
| CD-SJT Agency |  |  | -0.10 [-0.25; 0.06] |  |
| CD-SJT Communion |  |  |  | 0.00 [-0.16; 0.16] |
| Adjusted R² | **.055** | **.049** | **.058** | **.048** |
| ΔR² |  | -.006 | .003 | -.006 |

*Note*. Intercept always 0.00. 95% CI in parentheses. ΔR² in comparison to the Step 1 model. Gender was coded as 0 = male and 1 = female. Bold coefficients indicate significant results at *p* < .05.

**Table S2** Summary of Multiple Regression Analyses of Prediction on OSCE Interaction Performance for High-Stakes Sample

| Variable | Step 1 | Step 2a | Step 2b | Step 2c |
| --- | --- | --- | --- | --- |
| GPA | **0.19** [0.03; 0.34] | **0.20** [0.05; 0.36] | **0.18** [0.03; 0.34] | **0.17** [0.02; 0.32] |
| HAM-Nat | -0.01 [-0.16; 0.15] | -0.02 [-0.17; 0.14] | -0.01 [-0.17; 0.15] | -0.02 [-0.17; 0.14] |
| Gender | **0.17** [0.01; 0.33] | 0.15 [-0.01; 0.31] | **0.17** [0.01; 0.33] | **0.16** [0.00; 0.31] |
| HAM-SJT |  | 0.14 [-0.01; 0.29] |  |  |
| CD-SJT Agency |  |  | -0.06 [-0.21; 0.10] |  |
| CD-SJT Communion |  |  |  | **0.18** [0.03; 0.33] |
| Adjusted R² | **.057** | **.070** | **.054** | **.084** |
| ΔR² |  | .013 | -.003 | **.027** |

*Note.* Intercept always 0.00. 95% CI in parentheses. ΔR² in comparison to the Step 1 model. Gender was coded as 0 = male and 1 = female. Bold coefficients indicate significant results at *p* < .05.

**Controlling for Participating in Low-Stakes and High-Stakes Sample**

**Table S3** Descriptive Statistics and Correlations for Participants With Low-Stakes and High-Stakes Data

|  | *N* | *M* | *SD* | 1. | 2. | 3. | 4. | 5. | 6. | 7. | 8. | 9. | 10. | .11 |
| --- | --- | --- | --- | --- | --- | --- | --- | --- | --- | --- | --- | --- | --- | --- |
| 1. HAM-SJT LS | 66 | 0.65 | 0.18 | **-** |  |  |  |  |  |  |  |  |  |  |
| 1. HAM-SJT HS | 160 | 0.29 | 0.10 | **.27** | **-** |  |  |  |  |  |  |  |  |  |
| 1. CD-SJT Agency LS | 52 | 2.43 | 0.20 | **.36** | .26 | **-** |  |  |  |  |  |  |  |  |
| 1. CD-SJT Agency HS | 160 | 2.08 | 0.24 | -.07 | -.01 | .15 | **-** |  |  |  |  |  |  |  |
| 1. CD-SJT Communion LS | 54 | 2.58 | 0.19 | **.35** | -.06 | .07 | -.02 | **-** |  |  |  |  |  |  |
| 1. CD-SJT Communion HS | 160 | 2.69 | 0.21 | .10 | **.26** | .07 | **-.22** | .25 | **-** |  |  |  |  |  |
| 1. OSCE Interaction | 160 | 17.19 | 0.99 | **.25** | .14 (.20) | .19 | -.04 | -.14 | **.20** | **-** |  |  |  |  |
| 1. OSCE Other | 160 | 17.73 | 1.14 | .24 | .09  (.13) | .13 | .02 | -.12 | .10 | **.32** | **-** |  |  |  |
| 1. GPA | 160 | 23.98 | 3.60 | **-.28** | -.08 | .00 | -.01 | -.09 | .09 | **.22** | **.20** | **-** |  |  |
| 1. HAM-Nat | 160 | 0.72 | 0.15 | -.16 | .02 | .03 | -.03 | -.14 | .03 | -.05 | -.03 | -.05 | **-** |  |
| 1. Gender | 160 | 1.39 | 0.49 | .21 | .11 | .06 | .12 | .00 | .06 | **.20** | **.24** | **.19** | **-.21** | **-** |
| 1. Age | 160 | 21.00 | 2.60 | .18 | .04 | .03 | -.05 | .01 | -.02 | -.14 | -.02 | **-.66** | -.11 | -.12 |

*Note.* Correlations in parentheses were corrected for range restriction in the SJTs according to Thorndike’s Case 2 (Stauffer & Mendoza, 2001). Gender was coded as 0 = male and 1 = female. Bold coefficients indicate significant results at *p* < .05.

**Table S4** Summary of Multiple Regression Analyses of Prediction on OSCE Interaction Performance for High-Stakes Sample With SJT Data From Low-Stakes Instead of High-Stakes Participation

| Variable | Step 1 | Step 2a | Step 2b | Step 2c |
| --- | --- | --- | --- | --- |
| GPA | **0.19** [0.03; 0.34] | **0.18** [0.03; 0.34] | **0.18** [0.03; 0.34] | **0.18** [0.03; 0.33] |
| HAM-Nat | -0.01 [-0.16; 0.15] | -0.03 [-0.18; 0.13] | -0.02 [-0.17; 0.14] | -0.01 [-0.16; 0.15] |
| Gender | **0.17** [0.01; 0.33] | **0.14** [-0.02; 0.30] | 0.16 [0.00; 0.32] | **0.16** [0.00; 0.32] |
| HAM-SJT |  | 0.14 [-0.02; 0.29] |  |  |
| CD-SJT Agency |  |  | 0.06 [-0.10; 0.21] |  |
| CD-SJT Communion |  |  |  | **0.17** [0.02; 0.32] |
| Adjusted R² | **.057** | **.069** | **.055** | **.082** |
| ΔR² |  | .012 | -.003 | **.023** |

*Note.* Intercept always 0.00. 95% CI in parentheses. ΔR² in comparison to the Step 1 model. Gender was coded as 0 = male and 1 = female. Bold coefficients indicate significant results at *p* < .05.

**Controlling for Different CD-SJT Instructions in the High-Stakes Sample**

**Table S5** Descriptive Statistics and Correlations for Different CD-SJT Instructions

|  | *N* | *M* | *SD* | 1. | 2. | 3. | 4. | 5. | 6. | 7. | 8. | 9. | 10. |
| --- | --- | --- | --- | --- | --- | --- | --- | --- | --- | --- | --- | --- | --- |
| 1. HAM-SJT | 160 | 0.29 | 0.10 | **-** |  |  |  |  |  |  |  |  |  |
| 1. CD-SJT Agency Select | 97 | 2.07 | 0.22 | -.08 | **-** |  |  |  |  |  |  |  |  |
| 1. CD-SJT Agency Rank | 63 | 2.10 | 0.28 | .04 | **-** | **-** |  |  |  |  |  |  |  |
| 1. CD-SJT Communion Select | 97 | 2.64 | 0.20 | **.22** | -.19 | **-** | **-** |  |  |  |  |  |  |
| 1. CD-SJT Communion Rank | 63 | 2.77 | 0.19 | .21 | **-** | **-.35** | **-** | **-** |  |  |  |  |  |
| 1. OSCE Interaction | 160 | 17.19 | 0.99 | .14 (.20) | -.04 | -.04 | **.22** | .15 | **-** |  |  |  |  |
| 1. OSCE Other | 160 | 17.73 | 1.14 | .09  (.13) | -.04 | .09 | .14 | -.01 | **.32** | **-** |  |  |  |
| 1. GPA | 160 | 23.98 | 3.60 | -.08 | -.05 | .06 | .15 | -.02 | **.22** | **.20** | **-** |  |  |
| 1. HAM-Nat | 160 | 0.72 | 0.15 | .02 | .08 | -.19 | .03 | -.02 | -.05 | -.03 | -.05 | **-** |  |
| 1. Gender | 160 | 1.39 | 0.49 | .11 | -.04 | **.32** | .03 | .01 | **.20** | **.24** | **.19** | **-.21** | **-** |
| 1. Age | 160 | 21.00 | 2.60 | .04 | -.10 | .02 | -.04 | .03 | -.14 | -.02 | **-.66** | -.11 | -.12 |

*Note.* Participants received either an instruction to select one behavioral response corresponding most to their likely behavior or to rank all three behavioral responses according to the likelihood of showing this behavior. Participants with ranking instruction scored higher on CD-SJT Communion (*t*(137.39) = -4.14, *p* < .001) but not on Agency (*t*(110.24) = -0.72, *p* = .474)*.* Correlation in parentheses was corrected for range restriction in HAM-SJT according to Thorndike’s Case 2 (Stauffer & Mendoza, 2001). Gender was coded as 0 = male and 1 = female. Bold coefficients indicate significant results at *p* < .05.

**Table S6** Summary of Multiple Regression Analyses of Prediction on OSCE Interaction Performance for High-Stakes Sample With CD-SJT Instruction as Control Variable

| Variable | Step 1 | Step 2a | Step 2b | Step 2c |
| --- | --- | --- | --- | --- |
| GPA | **0.19** [0.03; 0.34] | **0.20** [0.05; 0.36] | **0.19** [0.03; 0.34] | **0.17** [0.02; 0.32] |
| HAM-Nat | -0.01 [-0.17; 0.14] | -0.02 [-0.17; 0.14] | -0.01 [-0.17; 0.14] | -0.02 [-0.17; 0.14] |
| Gender | 0.16 [0.00; 0.32] | 0.14 [-0.02; 0.30] | **0.17** [0.00; 0.33] | 0.16 [0.00; 0.32] |
| Instruction | 0.05 [-0.10; 0.21] | 0.03 [-0.13; 0.18] | 0.06 [-0.10; 0.21] | 0.00 [-0.16; 0.16] |
| HAM-SJT |  | 0.13 [-0.02; 0.29] |  |  |
| CD-SJT Agency |  |  | -0.06 [-0.21; 0.09] |  |
| CD-SJT Communion |  |  |  | **0.18** [0.02; 0.34] |
| Adjusted R² | **.054** | **.065** | **.051** | **.078** |
| ΔR² |  | .011 | -.002 | **.024** |

*Note.* Intercept always 0.00. 95% CI in parentheses. ΔR² in comparison to the Step 1 model. Instruction was coded as 0 = selecting and 1 = ranking. Gender was coded as 0 = male and 1 = female. Bold coefficients indicate significant results at *p* < .05.
